# Do Probiotics Cause a Shift in the Microbiota of Dental Implants—A Systematic Review and Meta-Analysis

**DOI:** 10.3389/fcimb.2022.823985

**Published:** 2022-03-16

**Authors:** Shariel Sayardoust, Anders Johansson, Daniel Jönsson

**Affiliations:** ^1^ School of Health and Welfare, Jönköping University, Jönköping, Sweden; ^2^ Department of Biomaterials, Institute of Clinical Sciences, Sahlgrenska Academy, University of Gothenburg, Gothenburg, Sweden; ^3^ Division of Molecular Periodontology, Department of Odontology, Umeå University, Umeå, Sweden; ^4^ Faculty of Odontology, Malmö University, Malmö, Sweden; ^5^ Department of Clinical Sciences, Lund University, Malmö, Sweden; ^6^ Department of Odontology and Oral Health, Public Dental Care of Skåne, Lund, Sweden

**Keywords:** probiotics, dental implant, microbiota, peri-implantitis, peri-implant mucositis

## Abstract

**Objective:**

The primary aim of this current systematic review and meta-analysis was to evaluate the potential microbiological effect of probiotics on the implant microbiota. The secondary aim was to evaluate if probiotics have any effect as an adjunct to non-surgical peri-implant treatment in reducing peri-implant mucositis and peri-implantitis clinical parameters—bleeding on probing, modified Gingival Index, and pocket depth.

**Methods:**

The research focus questions were constructed in accordance with the Participants, Intervention, Comparison, and Outcomes (PICO) criteria, and a PROSPERO protocol was registered. A comprehensive systematic search in MEDLINE *via* the PubMed, Scopus, and Web of Science Core Collection databases was conducted. Two independent reviewers screened the reports based on the PICO criteria—inclusion and exclusion criteria.

**Results:**

In total, 467 records were identified, and ultimately, 7 papers were included: 3 papers in the qualitative synthesis of microbiological effect and 4 in the meta-analysis synthesis on pocket depth. The data synthesis showed that probiotics had no detectable effect on the implant microflora, and in the following data synthesis, no clinical peri-implantitis variable showed a significantly beneficial effect from probiotics in the test group compared to the control group.

**Conclusion:**

Within the limitations of this review, the oral implant microflora is not affected by probiotics nor do probiotics add any effect to the conventional non-surgical treatment of peri-implant mucositis and peri-implantitis.

## Introduction

When replacing missing teeth, dental implants are a commonly used treatment option in modern dentistry, and osseointegration is the prerequisite of this treatment modality. Successful osseointegration involves a plethora of biological events, including inflammation, bone formation, and remodeling (Albrektsson and Johansson, 2001; Palmquist et al., 2010). Dental implants are a reliable treatment for rehabilitating both partly and completely edentulous areas, with high survival and success rates (Jung et al., 2012; Pjetursson et al., 2012) and associated with high patient satisfaction (Esposito et al., 1998; Sgolastra et al., 2015; Karlsson et al., 2020). However, technical and biological complications do occur, and the biological complications can be divided into peri-implant mucositis and peri-implantitis. The presence of an inflammatory lesion is a common denominator between the two conditions, although peri-implantitis is defined as bone resorption at the implant exceeding that of bone remodeling (Sanz and Chapple, 2012; Jepsen et al., 2015). According to the 2017 World Workshop on Classification of Periodontal and Peri-implant Diseases and Conditions, plaque is the etiological factor for peri-implant mucositis, with the main clinical characteristic being bleeding at gentle probing, and an increase in probing depth is often observed. Peri-implantitis is a plaque‐associated pathological condition occurring in tissues around dental implants and is characterized by progressive bone loss, bleeding on probing, and increased probing depth (Berglundh et al., 2018; Schwarz et al., 2018).

Under healthy conditions, there is an equilibrium between the implant microbiota and the host cells of the peri-implant tissues. At disease, the microbiota of the biofilm shift into dysbiosis, accompanied by an influx of pro-inflammatory cells in the peri-implant tissues. The impact of dysbiosis on peri-implantitis has been studied in both animal (Albouy et al., 2012; Fickl et al., 2015) and human models (Pontoriero et al., 1994; Zitzmann et al., 2001). The importance of the microbiota as an etiologic factor for both peri-implant mucositis and peri-implantitis would be further strengthened if a microbiological shift from probiotics could change the outcome of this progressive disease. Probiotics have a beneficial effect on gut microbiota and reduce the duration of antibiotic-associated diarrhea (Guo et al., 2019). Some studies suggest a beneficial effect from probiotics (Flichy-Fernández et al., 2015; Galofré et al., 2018), and this has been summarized in a meta-analysis (Silva et al., 2020) (although this analysis is in need of an update). The objective of the current review is to evaluate if probiotics cause a shift in the implant microbiota and if that shift is accompanied by improvements in the peri-implant mucositis and peri-implantitis clinical variables when compared to subjects receiving placebo. Specifically, this systematic review aimed to answer the following PICOS question: Does adding microbiota *via* probiotics alter the implant microbiota and/or improve the clinical parameters of peri-implant diseases, bleeding upon probing, pocket depth, or bone loss?

## Materials and Methods

### Protocol and Registration

The review protocol was developed and registered in the PROSPERO International Prospective Register of Systematic Reviews, hosted by the Centre for Reviews and Dissemination, University of York, National Institute for Health Research (United Kingdom) under the registration number CRD42020203298. The present manuscript follows the Preferred Reporting Items for Systematic Review and Meta-analyses statement for reporting a systematic review (Moher et al., 2009). Ethics approval was not required for this systematic review.

#### Focus Review Questions

Do probiotics cause changes in the microbiota at an implant or affect the clinical parameters such as bleeding upon probing, pocket depth, or bone loss?

### Eligibility Criteria

#### Inclusion Criteria

##### Population

Human subjects with oral implants replacing missing teeth were included in the study. There was no pre-selected cohort based on a specific risk factor or studies evaluating an implant system or implant components.

##### Intervention and Comparison

To investigate the impact of probiotics, the test group is administered with probiotics and the control group with placebo. Probiotics may be added to the conventional treatment of mucositis and peri-implantitis.

##### Outcomes

Changes in peri-implant microbiological composition (primary) and clinical variables (secondary) were the expected outcomes of this study.

##### Study Design

Clinical studies with a placebo control group and a test group given probiotics were included. Randomized clinical trials (RCTs) and cross-over studies are included as well.

#### Type of Outcomes

##### Primary Outcomes

Changes in microbiological composition (abundance of bacteria and/or diversity) at implants in the test group receiving probiotics *versus* the control group were considered primary outcomes. The data were summarized in a qualitative synthesis.

##### Secondary Outcomes

The secondary outcomes included changes in the clinical peri-implant variables bleeding on probing (BOP), modified Gingival Index (mGI) (Mombelli et al., 1987), and probing pocket depth in the test group receiving probiotics *versus* the control group. Preferably, all clinical variables can be summarized in a meta-analysis; however, if not all studies present BOP or mGI, then the bleeding variables will be presented in a qualitative synthesis.

### Information Sources and Search

A comprehensive systematic search was conducted for studies written in English in MEDLINE *via* the PubMed, Scopus, and Web of Science Core Collection databases. The final update was conducted on May 11, 2021. The terms used and the output are presented in **Table 1**. Eligibility assessment was performed through title and abstract evaluation and full-text analysis. Two reviewers screened the titles and abstracts (DJ and SS). The full text of potentially relevant studies was then obtained for independent assessment by the same reviewers to verify the fulfilment of the inclusion criteria. Any disagreement was resolved by a discussion between the reviewers. Two reviewers conducted all quality assessments independently.

**Table 1:** Search terms and output in the three MEDLINE databases *via* PubMed, Scopus, and Web of Science Core Collection.

Database	Search term			Output
PubMed *via* NLM	MeSH + all fields	1	“Peri-implantitis”[Mesh] OR peri-implant* OR peri implant* OR peri-implant* OR Prosth* OR mucosit*”	384,131
		2	“Probiotics” [Mesh] OR probiotic* OR prebiotic* OR “microbial dietary supplement*”	39,855
		3	Chemotherapy	3,562,085
		4	(1 AND 2) NOT 3	191
Scopus *via* Elsevier	Title–abstract–keywords	1	Peri-implant* OR peri implant* OR peri-implant* OR Prosth* OR mucosit*	14,760
		2	Probiotic* OR prebiotic* OR “microbial dietary supplement*”	69,306
		3	1 AND 2	27
Web of Science Core Collection (Clarivate)	(All fields)	1	Peri-implant* OR peri-implant* OR peri-implant* OR Prosth* OR mucosit*	201,857
		2	Probiotic* OR prebiotic* OR “microbial dietary supplement*”	55,963
		3	1 AND 2	227

* is part of the search-string.

The following data were extracted: (1) first author, (2) year of publication, (3) characteristics of the cohort—including sample size, mean age, sex ratio, and smoking habits, (4) definition used for mucositis and peri-implantitis, (5) follow-up time point(s), (6) duration of intervention, and (7) method used to analyze the microbiota.

#### Risk of Bias in Individual Studies

The recommendations of the Consolidated Standards of Reporting Trials statement were followed for the assessment of the methodological quality of the included RCTs. To determine and establish the validity of eligible trials, the authors used the Cochrane Handbook for Systematic Reviews of Interventions to assess the risk of bias (Higgins and Altman, 2008) associated with concealment of allocation, randomization, blinding of outcome assessor, and blinding of patients in the studies. There may be a risk of bias due to industry-sponsored studies. A funnel plot was constructed to analyze any skewness in the published reports in terms of positive or negative data. Grants and fees from companies are reported and discussed.

#### Summary Measures and Synthesis

The microbiological data was synthesized using qualitative synthesis. Clinical parameters were extracted and summarized in either qualitative synthesis or meta-analysis since all studies presented pocket depth, which was summarized in a meta-analysis. However, because some studies presented bleeding as BOP and some as mGI, bleeding was summarized in a qualitative synthesis.

Meta-analysis with random effects and funnel plot was performed in R ver 4.0.2 (package metaphor ver 2.4-0).

## Results

### Study Selection

**Figure 1:**
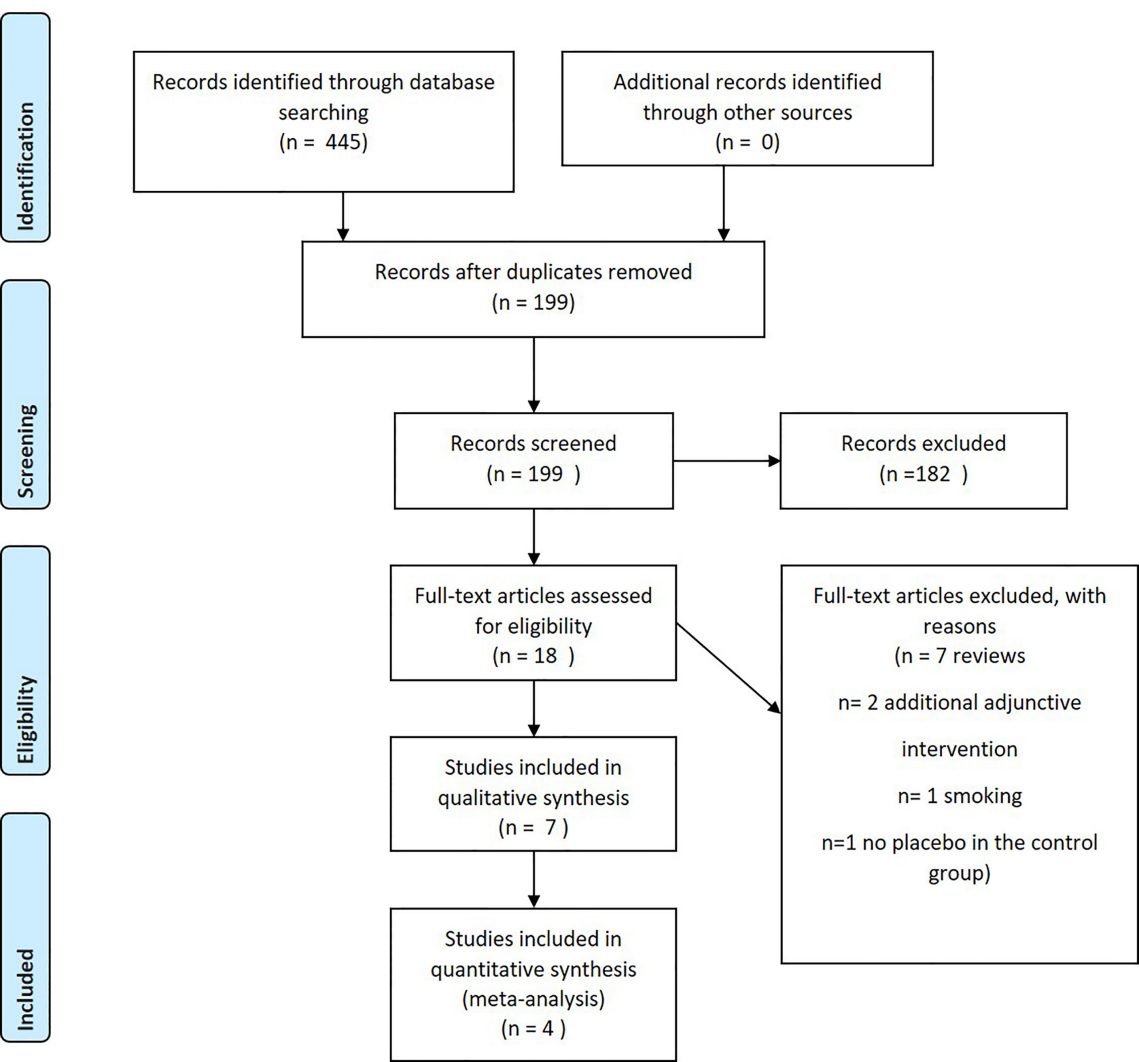
Preferred Reporting Items for Systematic Review and Meta-analyses flow diagram describing the selection process.

The selection flow chart is presented in **Figure 1**. A total of 467 records were identified. After the removal of duplicates, 199 remained. Eighteen full-text articles were assessed, and ultimately, 7 (Flichy-Fernández et al., 2015; Hallström et al., 2016; Galofré et al., 2018; Tada et al., 2018; Lauritano et al., 2019; Peña et al., 2019; Laleman et al., 2020) papers were included in the qualitative synthesis and 4 in the meta-analysis synthesis (Flichy-Fernández et al., 2015; Galofré et al., 2018; Peña et al., 2019; Laleman et al., 2020), as illustrated in **Figure 1B**.

### Study Characteristics

The study characteristics are summarized in **Table 2**. Seven intervention studies investigating the effects from probiotic supplements to the non-surgical treatment of peri-implant mucositis and peri-implantitis were included (Flichy-Fernández et al., 2015; Hallström et al., 2016; Galofré et al., 2018; Tada et al., 2018; Lauritano et al., 2019; Peña et al., 2019; Laleman et al., 2020). All studies were placebo-controlled, blinded, and randomized. One study is a crossover study (Flichy-Fernández et al., 2015). All studies examined non-surgical treatment that included oral hygiene instructions and scaling prior to the administration of probiotics or placebo. Tada et al. added azithromycin for 3 days prior to the administration of probiotics (Tada et al., 2018). Tada et al. and Peñja et al. included a regime of 15 days chlorhexidine (0.12%) rinsing prior to the probiotic/placebo administration protocol (Tada et al., 2018; Peña et al., 2019). In Laleman et al. (2020), the peri-implant pockets were treated with Air-N-Go Easy air polisher (Acteon). All studies used *Lactobacillus reuteri*, and the strain DSM 17938 and ATCC PTA 5289 were used in 6 studies (Flichy-Fernández et al., 2015; Hallström et al., 2016; Galofré et al., 2018; Tada et al., 2018; Peña et al., 2019; Laleman et al., 2020). In Lauritano et al. (2019), the strain was not specified.

**Table 2:** Characteristics of the studies included in the analysis of studies on probiotics.

Study	Study design	Participants (*N*)	Case definition	Age	Implants (*N*)	Smokers	Probiotics	Dosage	Time points	Plaque	Bleeding	Pocket depths	Bio-markers
Flichy-Fernández et al., 2015	Randomized, placebo-Controlled, double-blind, crossover	34 Group A (healthy) 22 Group B (M) 12	Healthy - PPD < 4 mm and no signs of inflammation. M - gingival redness, swelling, BOP, no bone loss	Group A (healthy) 63.6 ± 10.1 Group B M) 4 60.2 ± 7.4	77 Group A (healthy) 54 Group B (M) 23	Nonsmokers	*Lactobacillus reuteri* strains ATCC PTA 5289 and DSM 17938, 10⁸ CFU each strain	One tablet (probiotics/placebo)per day in 30 days	30 days Test or placebo. 6 months washout	Group A **Test Δ -0.59 ± 0.94 Control Δ 0.00 ± 0.82**	Group A **Test Δ -0.37 ± 0.73 Control Δ 0.44 ± 0.77**	Group A **Test Δ -0.16 ± 0.84 Control Δ 0.27 ± 0.72**	IL 1b, **IL 6 and IL 8**
										Group B **Test Δ -0.74 ± 1.05 Control Δ 0.00 ± 1.28**	Group B Test Δ -0.09 ± 1.08 Control Δ 0.48 ± 0.79	Group B **Test Δ -1.09 ± 0.90 Control Δ 0.18 ± 0.65**	
Galofré et al., 2018	Randomized, placebo-controlled, triple-blind, parallel-design	44 M: 22: 11 Test, 11 Control P: 22: 11 Test, 11 Control	M - BoP and/or suppuration and no bone loss. P - BoP and/or suppuration, PPD ≥5 mm and bone loss of ≥2 and/or ≥3 mm implant threads-	M Test 61.5 ± 10.4 Control 60.0 ± 9.5 P Test 61.7 ± 7.0 Control 56.8 ± 9.3	M: 22: 11 Test, 11 Control P: 22: 11 Test, 11 Control	Nonsmokers	*Lactobacillus reuteri* strains ATCC PTA 5289 and DSM 17938, 10⁸ CFU each strain	One tablet (probiotics/placebo)per day in 30 days	BL, 30 and 90 days	M Test Δ −0.16 ± 0.17 Control Δ −0.09 ± 0.04	M Test Δ −32% ± 0.24* Control Δ −7% ± 0.24	M Test Δ −0.48 ± 0.50 Control Δ −0.15 ± 0.36	
										P Test Δ −0.16 ± 0.09 Control Δ −0.10 ± 0.11	P Test Δ −20% ± 0.22 Control Δ −10% ± 0.18	P **Test Δ −0.55 ± 0.37 Control Δ −0.20 ± 0.35**	
Laleman et al., 2020	Randomized, placebo-controlled, double-blind	19 Test 9 (5 male/4 female) Control 10 (4 male/6 female)	P- PPD ≥4 mm with BOP, bone loss of > 1 mm	Test 64 ± 11 Control 69 ± 9	19 Test 9 Control 10	Nonsmokers	*Lactobacillus reuteri* strains ATCC PTA 5289 and DSM 17938, 10⁸ CFU each strain	1.9 ± 0.3 tablets per day in Test group and 1.6 ± 0.4 in Control group. Duration not specified.	BL, 6-, 12- and 24-week	**Test Δ −13 ± 14%**	Test Δ −27% ± 23 Δ −0.93 ± 0.67	Test Δ −1.02 ± 0.69	
										**Control Δ −2 ± 16%**	Control Δ −33% ± 27 Δ −0.56 ± 0.97	Control Δ −1.27 ± 1.00	
Peña et al., 2019	Randomized, placebo-controlled, triple-blind	50 Test 25 Control 25	M - BOP with gingival redness, swelling, no bone loss	Test 55.96 ± 10.81 Control 61.16 ± 10.62	50 Test 25 Control 25	Test 0% Control 4%	*Lactobacillus reuteri* strains ATCC PTA 5289 and DSM 17938, 10⁸ CFU each strain	One tablet (probiotics/placebo)per day in 30 days	BL, 15, 45, and 135 days	Test Δ −48.0% Control Δ −44.0%	Test Δ 36.0% Control Δ 40.0%	Test Δ − 0.21 ± 0.48	
												Control Δ − 0.34 ± 0.50	
Hallström et al., 2016	Randomized, placebo-controlled, double-blind	49 Test 24 Control 25	M- PPD > 4 mm with BOP and/or suppuration	24–85 years Test 53.7 (19.6) Control 63.3 (17.2)	49 Test 24 Control 25	Test 29% Control 8%	*Lactobacillus reuteri* strains ATCC PTA 5289 and DSM 17938, 10⁸ CFU each strain	One tablet (probiotics/placebo) twice daily for 3 months	BL, 1, 2, 4, 12 and 26 weeks	Test BL 26% 26w 26%	Test BL 54% 26w 14%	Test BL 4.3 ± 1.1 26w 3.7 ± 1.3	IL-1b, IL-1RA, IL-4, IL-6, IL-8, IL-17A, CCL5, TNF-a, IFN-g and GMCSF
										Control BL 32% 26w 15%	Control BL 58% 26w 17%	Control BL 4.0 ± 1.4 26 w 3.5 ± 1.5	
Tada et al., 2017	Randomized, placebo-Controlled, double blind	30 Test 15 Control 15	Mild to moderate P - PPD > 4 and < 7 mm, BOP or suppuration and >2 mm bone loss	Test 68.80 ± 7.46 Control 65.87 ± 8.84	30 Test 15 Control 15	Test 20% Control 7%	*Lactobacillus reuteri* strains ATCC PTA 5289 and DSM 17938, 10⁸ CFU each strain	One tablet a day for 6 months	0, 4, 12 and 24 weeks	Test 0w 1.07 ± 0.7 24w 1.13 ± 0.74	Test 1.27 ± 0.70 24w 0.93 ± 0.79	Test 0w 3.64 ± 0.83 24w 3.21 ± 0.84	
										Control 0w 1.27 ± 1.03.74 24w 1.20 ± 0.68	Control 0w 1.40 ± 0.91 24w 1.53 ± 0.92	Control 0w 3.53 ± 0.97 24w 3.47 ± 0.95	
Lauritano et al., 2019	Randomized placebo-Controlled	10	P - definition not specified	No data available	10	No data available	*Lactobacillus reuteri*, strain not specified	One tablet (probiotic or placebo) for 4 weeks	BL and 28 days	No data available	No data available	No data available	

The bold font and asterisk indicate significance between baseline and latest timepoint and test and control, respectively. Data on bleeding presented in % refer to bleeding on probing and otherwise modified Gingival Index.

Group A, healthy; Group B, peri-implant mucositis; M, mucositis; P, peri-implantitis; IL-1β, interleukin- 1β; IL-6, interleukin-6; IL-8, interleukin-8; IL-1RA, interleukin-1 receptor antagonist; IL-4, interleukin-4; IL-7A, interleukin-7A; CCL-5, chemokine ligand-5; IFN-g, interferon gamma; GMCSF, granulocyte colony-stimulating factor.

### Qualitative Synthesis

**Figure 2:**
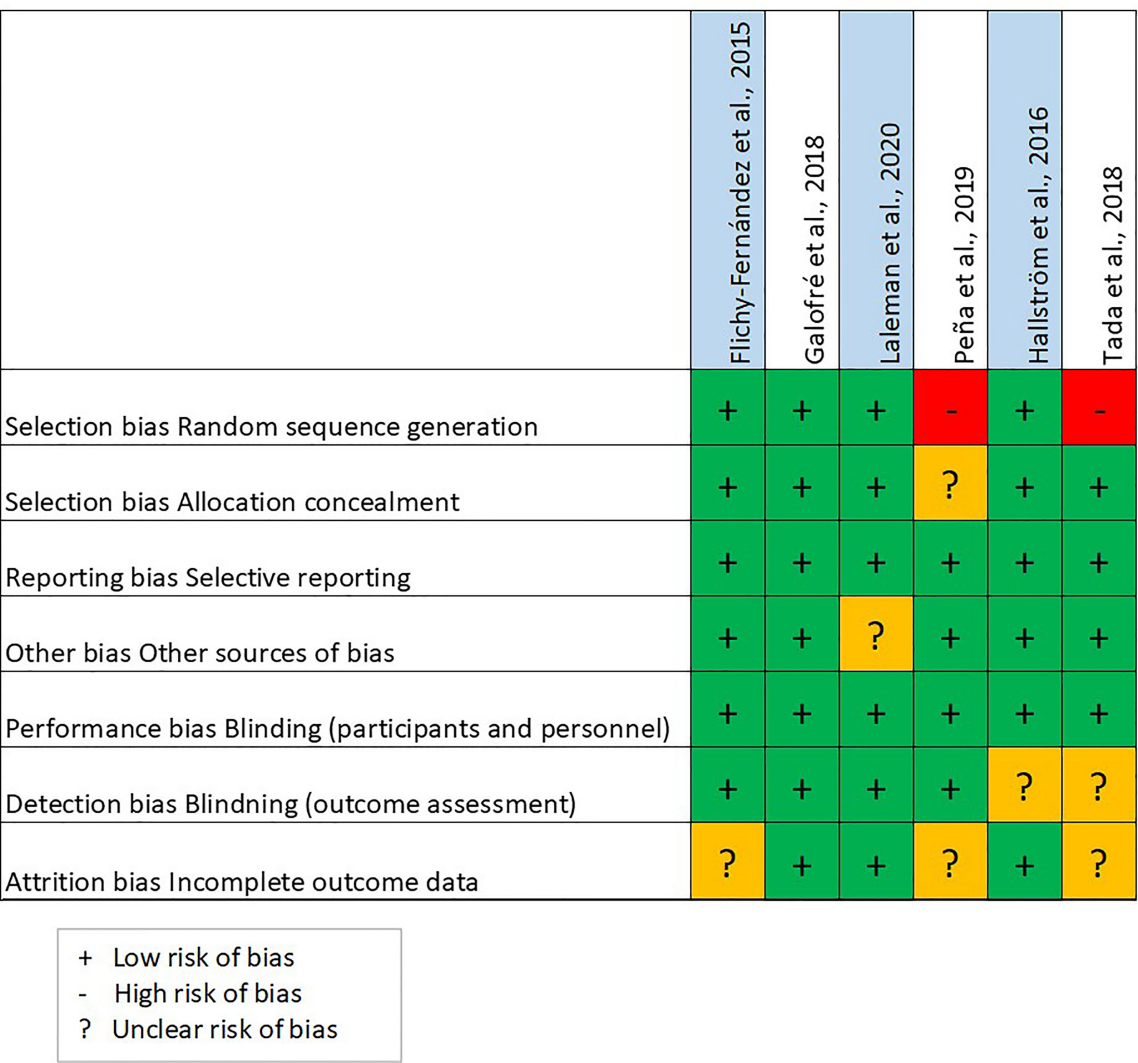
Risk of bias.

The results regarding risk of bias, using the Cochrane Handbook for Systematic Reviews of Interventions to assess the risk of bias, are illustrated in **Figure 2**. Four out of the 7 studies revealed low bias (Flichy-Fernández et al., 2015; Hallström et al., 2016; Galofré et al., 2018; Laleman et al., 2020). Selection bias was noted in the remaining 3 studies (Tada et al., 2018; Lauritano et al., 2019; Peña et al., 2019).

Out of the 7 probiotic intervention studies included, 6 investigated changes in the microflora composition (Flichy-Fernández et al., 2015; Hallström et al., 2016; Galofré et al., 2018; Tada et al., 2018; Lauritano et al., 2019; Peña et al., 2019; Laleman et al., 2020). Hallström et al. (2016) studied the microbiota using checkerboard DNA–DNA hybridization technique targeting 12 bacterial strains. Galofré et al. (2018) and Peña et al. (2019) used real-time (rt)PCR targeting A*ggregatibacter actinomycetemcomitans*, *Tannerella forsythia*, *Porphyromonas gingivalis*, *Treponema denticola*, *Prevotella intermedia*, *Peptostreptococcus micros*, *Fusobacterium nucleatum*, *Campylobacter rectus*, and *Eikenella corrodens*; Laleman et al. (2020) qPCR-targeted *P. gingivalis*, *P.intermedia*, *F. nucleatum*, and *A. actinomycetemcomitans*. Lauritano et al. (2019) real-time PCR-targeted *P. gingivalis*, *T. forsythia*, and *T. denticola*; Tada et al. (2018) targeted *F. nucleatum*, *P. gingivalis*, *P. intermedia*, *A. actinomycetemcomitans*, *T. denticola*, and *T. forsythia* using a multiplex rt-PCR invader assay. There was only a limited difference between the probiotic and placebo groups in terms of microbiological abundance (see **Table 3**). Only Galofré et al. (2018) reported an inter-group difference of abundance of *P. gingivalis* (*p* = 0.031). Looking at all the included studies, there is no conclusive data confirming the presence of differences in the implant microbiota that were caused by adding probiotics.

**Table 3:** Quantitative synthesis of the microbiological outcome of the included studies.

Study	Detection method	Microbial targets	Microbial findings inter group
Galofré et al., 2018	qPCR	Periodontal pathogens	*P. gingivalis*
Laleman et al., 2020	qPCR	Periodontal pathogens	No differences
Peña et al., 2019	qPCR	Periodontal pathogens	No differences
Hallström et al., 2016	DNA-DNA checkerboard	Periodontal pathogens	No differences
Tada et al., 2018	qPCR	Periodontal pathogens	No differences
Lauritano et al., 2019	qPCR	Periodontal pathogens	No differences

qPCR, quantitative PCR.

### Synthesis of Additional Outcome

The 7 studies by Flichy-Fernandez et al., Galofré et al., Hallström et al., Laleman et al., Lauritano et al., Pena et al., and Tada et al. (Flichy-Fernández et al., 2015; Hallström et al., 2016; Galofré et al., 2018; Tada et al., 2018; Lauritano et al., 2019; Peña et al., 2019; Laleman et al., 2020) that evaluated the effects of probiotic supplements in adjunct to the conventional management of peri-implant mucositis and peri-implantitis are summarized in **Table 2**. The included studies (Flichy-Fernández et al., 2015; Hallström et al., 2016; Galofré et al., 2018; Tada et al., 2018; Lauritano et al., 2019; Peña et al., 2019; Laleman et al., 2020) had a duration interval of 30 days to 26 weeks. Most of the studies used *L. reuteri* with the same strain and a similar dosage of 1 tablet/lozenge a day for approximately 1 month (Flichy-Fernández et al., 2015; Galofré et al., 2018; Lauritano et al., 2019; Peña et al., 2019; Laleman et al., 2020). In 2 studies by Hallström et al. and Tada et al. (Hallström et al., 2016; Tada et al., 2018), the duration of the intake of probiotics/placebo was longer at 6 months and 12 weeks, respectively.

Galofré et al. detected a beneficial effect of probiotics on BOP (Galofré et al., 2018), while Flichy-Fernandez et al. only detected effects on plaque index and probing pocket depth reduction (Flichy-Fernández et al., 2015). Overall small differences in clinical parameters were detected in the respective studies from probiotic intervention; however, these results were not reproduced in the other studies.

Hallström et al. (2016) and Flichy-Fernández et al. (2015) also evaluated the immunological effects of probiotics in peri-implant crevicular fluid. Hallström et al. reported no effects from probiotics on a large array of cytokines, and while Flichy-Fernández et al. reported no effect on IL-1β, they did report lower levels of IL-6 and IL-8 in the test group compared to the placebo group.

**Figure 3:**
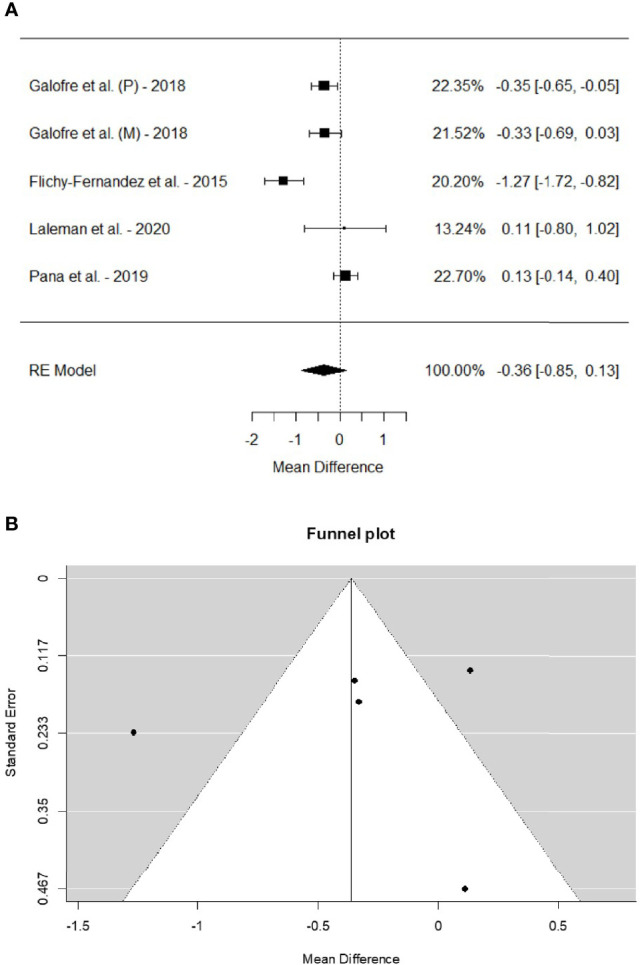
**(A)** Forest plot on the difference between the test and placebo groups in mean change in probing pocket depth before and after intervention. **(B)** Funnel plot depicting the publication bias for the studies included in the meta-analysis.

Four out of the 7 studies included were eligible to be analyzed in a meta-analysis on changes in pocket depth from the adjunctive probiotic intervention presented in **Table 4** and **Figure 3B**. The studies treating mucositis (Flichy-Fernández et al., 2015; Galofré et al., 2018; Peña et al., 2019) and peri-implantitis (Galofré et al., 2018; Laleman et al., 2020) were merged. In Galofré et al. (2018), the 2 cohorts with mucositis and peri-implantitis were treated separately. Laleman et al. presented the treatment outcome at 3 and 6 months. Here the earlier time-point was chosen in order to minimize the inter-study variance (Laleman et al., 2020). The model resulted in a total weighted pooled mean attenuation in probing depth of -0.36 mm (95% confidence interval, -0.85–0.13) from adding probiotics to the conventional treatment. However, as indicated by the confidence interval, the result did not reach statistical significance.

**Table 4:** Changes in probing pocket depth in the control and test groups in the studies included in the meta-analysis in **Figure 3**.

Study	P/M	n Ctr	Delta Mean Ctr	Delta SD Ctr	n Test	Delta Mean Test	Delta SD Test	Intervention time
Galofré et al., 2018	P	11	-0.2	0.35	11	-0.55	0.37	3 m
Galofré et al., 2018	M	11	-0.15	0.36	11	-0.48	0.5	3 m
Flichy-Fernández et al., 2015	M	23	0.18	0.65	23	-1.09	0.9	1 m
Laleman et al., 2020	P	10	-1.15	1.0	9	-1.04	1.03	3 m
Peña et al., 2019	M	25	-0.34	0.5	25	-0.21	0.48	4.5 m

The studied cohorts had either peri-implantitis (P) or mucositis (M). Delta refers to follow up - baseline.

### Risk of Bias Across Studies and in Individual Studies

The funnel plot for the meta-analysis forest plot is presented in **Figure 3B**.

The company Sunstar Suisse in Switzerland sell probiotic products manufactured by BioGaia AB in Sweden. All studies on probiotics, except that of Lauritano et al. (2019), were provided with free samples from BioGaia for the studies. Peña et al. (2019) declare that their study was partly supported by a research grant from Sunstar Suisse. Hallström et al. (2016) declared that the last author has received grants for a PhD student from BioGaia AB, and Laleman et al. (2020) declared that the last author has received lecturing fees from BioGaia AB.

## Discussion

The focus review question of this systemic review is whether there are changes in the microbiological composition at implants in the test group receiving probiotics versus that in the placebo-controlled group. The qualitative synthesis showed no inter-group difference in implant microbiota resulting from probiotic treatment. In terms of the clinical outcome probing pocket depth, the probiotic treatment did not cause any additional beneficial treatment effect to the standard treatment compared to placebo in a meta-analysis.

Due to the heterogeneity in measuring mucosal bleeding, mGI, and BOP, these important indicators of peri-implant disease could not be summarized in a meta-analysis forest plot. The analysis of probing depth allowed the inclusion of 4 studies in a meta-analysis forest plot that reported the mean and standard deviation change in probing depth. The 4 studies in the forest plot included interventions in both peri-implant mucositis and peri-implantitis, which led to heterogeneity in the analysis compared to a separate meta-analysis on peri-implant mucositis and peri-implantitis. However, a separate analysis would have reduced the power of the analysis, and probing pocket depth is an important disease-associated clinical variable in both peri-implant mucositis and peri-implantitis (Berglundh et al., 2018).

A funnel plot, shown in **Figure 3B**, was constructed to visualize any publication bias. In the funnel plot, the studies should ideally be distributed within the funnel, which is not the case in **Figure 3B**. However, the studies outside the plot are evenly distributed on both sides of the funnel.

In terms of bias due to financial support, the companies Sunstar Suisse and BioGaia have sponsored some of the included studies by providing the products administered to the study subjects. Those same companies have also provided grants and fees for some of the included studies. This would have been an issue if the results from any of the sponsored studies were to stand out from the rest of the studies in the funnel plot; however, that is not the case in our plot.

Selection bias seems to be a concern with regards to some of the studies as shown by the bias assessment performed using the Cochrane Handbook for Systematic Reviews of Interventions. This should, of course, be considered when drawing conclusions from this systematic review.

Another limitation of all included studies is the choice of microbiota analysis method. When the earlier studies were performed, 16S sequencing technique was not available. This technique is superior at detecting any changes in relative abundance and diversity in the microbiological community when compared to techniques that require a pre-selection of specific species associated with periodontal disease, such as checkerboard DNA–DNA hybridization and rt-PCR. Hence, it cannot be ruled out that the lack of changes in the microbiota composition after a probiotic treatment could be due to the method not being able to detect that difference.

A systemic meta-analysis has recently been published on the subject of probiotics and peri-implantitis disease parameters (Silva et al., 2020). In the meta-analysis of that paper, the authors have unfortunately compared the means of the later time-point in the test and control group regardless of the baseline means. Here the delta means (mean from later time-point - baseline mean) were compared in the placebo and test groups, which allows for differences in baseline levels.

The systematic review by Silva et al. (2020) included the paper by Alquatani et al. (2019), in which probiotics were evaluated in a group of never-smokers and a group of current smokers. To include this study would have violated the present PICO criteria in terms of the specified population. In a recent paper from the same group (Alqahtani et al., 2021), the control group did not receive placebo, and the delta means and standard deviations were not presented, which was a requirement in the meta-analysis.

An additional consideration for the probiotic studies that were evaluated in the present review is that they all used a strain of *L. reuteri* (24-30). The original target of *L. reuteri* probiotic treatment is the potential to interfere with the gut microbiota and to improve the integrity of the intestinal mucosa (Judkins et al., 2020). Accumulated data suggest that the microbiota may affect the host through the production of bioactive microbial metabolites that act as signaling messengers *via* penetration of the host’s blood circulation and tissues (Koh and Bäckhed, 2020). In the probiotic treatment strategy for peri-implantitis, a positive effect on remission might involve systemic interactions from the gut microbiota. It may be speculated that a probiotic strain residing from the commensal oral microflora may be more suitable for achieving a direct change in the oral microbiota.

The current systemic meta-analysis review has several limitations. The number of studies included in the analysis is relatively small, and so is the size of the studied cohorts, causing a wider spread of the results. In the probiotic trials, the number needed to treat is high due to the great heterogeneity in the individual microbiota setup. One might also question if randomized clinical trials are the best study designs to use when evaluating probiotics (Zeilstra et al., 2018); hence, randomized clinical trials were not included in the inclusion criteria of the current review. According to Zeilstra et al. (2018), it should be taken into consideration that there is a general shortcoming in all studies involving probiotic and prebiotic interventions. Whether or not a patient is susceptible to interventions is, to a large extent, dependent on the patient’s microbiota and nutrients, and it is a great challenge to randomize this in a controlled manner. In the future, it would be advantageous if it were possible to establish, perhaps through the use of specific biomarkers of a metabolic or proteomic character, which individuals are susceptible to probiotics for inclusion in a study. There is a great need for more stringent protocols and larger study cohorts to further evaluate the effect of probiotic-based intervention. The combined administration of prebiotic nutrients together with the probiotic bacteria might contribute to a prolonged survival of the supplemented bacteria (Adamberg et al., 2014). With respect to these circumstances, data extracted from the studies evaluated in the current systematic review are of limited impact for the research topic.

In terms of grading evidence, the review did not find any studies to support any of our focus questions. Based on the synthesis of additional outcomes, the review concludes that the use of adjunctive probiotics when treating peri-implantitis is not to be considered as evidence-based care.

This systemic review altogether shows that probiotics neither alter the implant microbiota nor add any clinically beneficial effect to the standard treatment of peri-implant mucositis and peri-implantitis. Based on the currently available literature, probiotics should not be recommended as an adjunct in the non-surgical treatment of mucositis or peri-implantitis. In future studies, a global microbiomics approach may be suitable when evaluating microbiological shifts. It may also be interesting to target subjects with a previous beneficial experience of probiotics in future trials.

## Author Contributions

SS and DJ provided substantial contributions to the conception and design of the study and in the acquisition, analysis, and interpretation of data. AJ contributed to the conception and design of the study and interpretation of data for the work. SS, AJ, and DJ drafted the work and revised it critically for important intellectual content, gave final approval of the version to be published, and agreed to be accountable for all aspects of the work in ensuring that questions related to the accuracy and integrity of any part of the work are appropriately investigated and resolved. DJ conceived and planned the analysis and manuscript. All authors contributed to the article and approved the submitted version.

## Funding

FUTURUM, the Academy of Health and Care, unit of Research and Development, Jönköping County.

## Conflict of Interest

The authors declare that the research was conducted in the absence of any commercial or financial relationships that could be construed as a potential conflict of interest.

## Publisher’s Note

All claims expressed in this article are solely those of the authors and do not necessarily represent those of their affiliated organizations, or those of the publisher, the editors and the reviewers. Any product that may be evaluated in this article, or claim that may be made by its manufacturer, is not guaranteed or endorsed by the publisher.
